# Transcriptomic Networks Reveal the Tissue-Specific Cold Shock Responses in Japanese Flounder (*Paralichthys olivaceus*)

**DOI:** 10.3390/biology12060784

**Published:** 2023-05-28

**Authors:** Jiayi He, Qing Zhu, Ping Han, Tianyu Zhou, Juyan Li, Xubo Wang, Jie Cheng

**Affiliations:** 1 Key Laboratory of Marine Genetics and Breeding (Ocean University of China), Ministry of Education, 5 Yushan Road, Qingdao 266003, China; 2 Key Laboratory of Tropical Aquatic Germplasm of Hainan Province, Sanya Oceanographic Institution, Ocean University of China, Sanya 572024, China; 3 Key Laboratory of Aquacultural Biotechnology (Ningbo University), Ministry of Education, 169 Qixingnan Road, Ningbo 315832, China; 4 Laboratory for Marine Fisheries Science and Food Production Processes, National Laboratory for Marine Science and Technology (Qingdao), 1 Wenhai Road, Qingdao 266237, China

**Keywords:** cold shock stress, tissue-specific response, coexpression network, cell cycle, *Paralichthys olivaceus*

## Abstract

**Simple Summary:**

Low temperature is an often overlooked stress that many fish face due to both natural and anthropogenic causes. Japanese flounder (*Paralichthys olivaceus*) is an economically important aquaculture fish species broadly cultivated in east Asia, mainly along the coast of the Bohai and Yellow Seas in China, as well as the coast of Korea and east Japan. Natural and cultivated *P. olivaceus* may suffer from cold stress during the winter months. In this study, modulated transcriptomic responses to 10 °C acute cold stress were investigated in the gills, hearts, livers, and spleens of *P. olivaceus*. Based on transcriptome and weighted gene coexpression network analysis, tissue-specific cold responsive modules (CRMs) were identified, which revealed a cascade of specific cellular responses to cold stress in different tissues. Our results illustrate the diverse and modulated regulation of the cellular process and stress response to low temperature, which provide essential insights for the conservation and cultivation of *P. olivaceus* in cold water.

**Abstract:**

Low temperature is among the important factors affecting the distribution, survival, growth, and physiology of aquatic animals. In this study, coordinated transcriptomic responses to 10 °C acute cold stress were investigated in the gills, hearts, livers, and spleens of Japanese flounder (*Paralichthys olivaceus*), an important aquaculture species in east Asia. Histological examination suggested different levels of injury among *P. olivaceus* tissues after cold shock, mainly in the gills and livers. Based on transcriptome and weighted gene coexpression network analysis, 10 tissue-specific cold responsive modules (CRMs) were identified, revealing a cascade of cellular responses to cold stress. Specifically, five upregulated CRMs were enriched with induced differentially expressed genes (DEGs), mainly corresponding to the functions of “extracellular matrix”, “cytoskeleton”, and “oxidoreductase activity”, indicating the induced cellular response to cold shock. The “cell cycle/division” and “DNA complex” functions were enriched in the downregulated CRMs for all four tissues, which comprised inhibited DEGs, suggesting that even with tissue-specific responses, cold shock may induce severely disrupted cellular functions in all tissues, reducing aquaculture productivity. Therefore, our results revealed the tissue-specific regulation of the cellular response to low-temperature stress, which warrants further investigation and provides more comprehensive insights for the conservation and cultivation of *P. olivaceus* in cold water.

## 1. Introduction

Temperature, as the major environmental stimulating factor, has essential effects on almost all biological processes in organisms, affecting their physiological and metabolic activities, immune and antioxidative responses, growth and behavior, as well as migration, especially for poikilothermic teleost species [1,2]. In the context of global climate change, a large number of studies on thermal stress in fish have mainly focused on high-temperature challenge in conservation and fishery management [1]; compared with heat stress, low-temperature exposure can also frequently occur in aquatic environments as the result of nature and/or anthropogenic causes [2].

A sharp reduction in ambient temperature is defined as cold shock, which has the potential to cause a rapid decline in body temperature, resulting in a cascade of physiological and behavioral responses [3]. Teleosts have evolved biochemical and physiological adaptations to low-temperature challenges. A conservative responsive mechanism exists in different fish species under chronic long-term low-temperature and acute short-term cold stress. For example, cold stress can result in the increase in endogenous reactive oxygen species (ROS) [4]. ROS generation may further contribute to DNA damage and apoptosis [5], which can help to maintain cellular homeostasis and play vital roles in the immune response [6,7]. Moreover, cold stress can also affect the survival of teleosts, cause mass mortality in aquaculture, and has important impacts on reproductive, metabolic, and developmental functions in teleosts [3].

In addition, many studies have pointed out that tissues involved in the physiological response and acclimation to low-temperature stress can exhibit tissue-specific characteristics [8,9,10]. For example, fish gills are directly exposed to aquatic environment, are the target for the temperature stress response, and are involved in many physiological processes, including respiration, ion- and osmo-regulation, immune response, and the acid–base balance [11]. The heart supplies power for the circulatory system to maintain the survival of the organism [12]. The liver plays an essential role in the metabolism, storage, and distribution of carbohydrates, proteins, and lipids in fish [13]. The spleen is mainly composed of lymph nodes and plays important roles in the hematopoietic, immune, and blood storage of fish [13]. Moreover, there are many functional pathways, including mitochondrial function, antioxidation, apoptosis, carbohydrate/lipid metabolism, DNA/RNA processing, and protein catabolism, that show temperature-dependent regulation [14,15,16,17]. Therefore, investigating the molecular mechanisms among the various tissues of fish that are affected by low temperature could provide a better understanding of how organisms adapt or respond to aquatic environmental challenges.

Recently, with the increasing abundance of sequencing data, weighted gene coexpression network analysis (WGCNA) is a powerful method that has been used to provide a comprehensive understanding of gene interaction networks. WGCNA focuses on the modulated regulation of the full-complement gene sets that comprise complex networks, transitioning data analysis from being gene-centric to systematically network-centric [18]. Therefore, the combination of transcriptome and network analysis will provide insightful implications about the tissue-specific responses of fish to dynamic environmental stresses through the functioning of cellular signaling pathways [19].

Japanese flounder (*Paralichthys olivaceus*) is an economically important aquaculture teleost species broadly cultivated in east Asia, mainly along the coast of the Bohai and Yellow Seas in China, as well as along the coast of Korea and east Japan [14]. Many studies have illustrated the effect of heat stress on the mechanisms of sexual differentiation, energy metabolism, neurosecretion, and stress-related gene regulation in *P. olivaceus* [14,20,21,22,23]. However, as a temperate water fish, nowadays, temperature decline has also influenced the survival, growth, distribution, and reproduction of this species [24]. For example, in the natural habitat of *P. olivaceus,* there are several months of cold winter, with the lowest temperature being 0 °C [25], and a seasonal water mass, the Yellow Sea Cold Water Mass, appears during the summer half of the year in the central bottom water with low temperature (<10 °C) [26], which makes the *P. olivaceus* growth rate significantly decrease and reduces aquaculture productivity [25,27]. To illustrate the tissue-specific regulation under cold stress, in this study, *P. olivaceus* specimens were subjected to 10 °C acute cold shock stress to observe the cellular damage to the gill, heart, liver, and spleen tissues, and transcriptome data were comprehensively analyzed with WGCNA. The results present a fundamental reference for cold shock damage in different *P. olivaceus* tissues and provide vital insights for the conservation and cultivation of *P. olivaceus* in cold water.

## 2. Materials and Methods

### 2.1. Acute Cold Shock Challenge for Adult P. olivaceus

A total of 18 adult *P*. *olivaceus* specimens (average body weight 866 ± 166 g, average total length 33 ± 4.5 cm) were obtained from Nanshan market, Qingdao, China, and acclimated in seawater in a laboratory aquarium (18 °C, 14:10 h light:dark, 30 ppt salinity) for one week. The seawater was aerated and refreshed once per day. Then, the *P*. *olivaceus* specimens were randomly divided into two groups: a control group under a temperature of 18 °C, and an acute cold shock group, which were abruptly transferred to 10 °C sea water for a short term of 6 h. There were 9 specimens in each group with 3 individuals in each of the 3 tanks as replicates, and no fish died during the experiment. Circulating water refrigerators (RESUN, Shenzhen, China, CL650, 650 W, 1/4HP) were employed to maintain the cold temperature. After the cold shock challenge, *P*. *olivaceus* specimens were euthanized with MS-222, and the gill, heart, liver and spleen tissues from each specimen were sampled and stored at −80 °C with lipid nitrogen for RNA isolation and in Bouin’s fluid for histological observation.

### 2.2. Histological Examination of P. olivaceus Tissues

To observe the histological changes under cold stress, the *P*. *olivaceus* tissues from Bouin’s fluid were dehydrated following a successive ethanol gradient of 50%, 70%, 90%, 95%, and 100%. Then, the tissue samples were transparentized with xylene and ethanol mixture, embedded into paraffin, and sliced into a thickness of 5 µm. The samples were further dewaxed and stained with hematoxylin and eosin (H&E) (Solarbio, Beijing, China) following the traditional method [14]. Finally, the samples, which were sealed with neutral gum, were observed and photographed with a Nikon Eclipse TiU microscope (Nikon, Tokyo, Japan) for histological examination.

### 2.3. RNA Isolation, Library Preparation, and Sequencing

The gill, heart, liver, and spleen tissues of *P. olivaceus* were sampled and frozen with liquid nitrogen. Total RNA was isolated using a method with SparkZol Reagent (SparkJade, Jinan, China). The genomic DNA contamination was removed by RNase-free DNase I (TaKaRa, Beijing, China) treatment. The total RNA was then qualified and quantified by 1.5% agarose gel electrophoresis and spectrophotometry, respectively. The RNA samples were further employed for RNA sequencing library preparation and high-throughput sequencing. The RNA sequencing was conducted on an Illumina Hiseq 4000 platform at the Beijing Novogene company (Novogene, Beijing, China).

### 2.4. Transcriptome Analysis of P. olivaceus Tissues in Response to Cold Shock Stress

To investigate the gene expression profiles of *P. olivaceus* under cold shock stress, 24 transcriptome datasets from four tissues (gill, heart, liver, and spleen) of both the control and cold shock groups were analyzed. After trimming adaptors and removing low-quality reads, the clean reads were mapped against the *P*. *olivaceus* genome using the Hisat and StringTie pipeline [28] with default parameters. The fragments per kilobase of exon per million mapped reads (FPKM) values were used to estimate gene expression levels with StringTie [28]. Differentially expressed genes (DEGs) between the control group and the cold shock group were analyzed using edgeR, with a threshold *q*-value of <0.05 and |log_2_FoldChange(FC)| > 1.5. The R package clusterProfiler 4.0 was employed for Gene Ontology (GO) functional enrichment analysis. TBtools [29] was utilized to draw heatmaps with the log_2_FC values.

### 2.5. Gene Coexpression Network Construction and Functional Characterization

WGCNA [18] was conducted to characterize the modulated gene interaction patterns across different *P. olivaceus* tissue samples with WGCNA R library [18]. A gene dendrogram was employed to identify modules using the dynamic tree cut method (minimum module size = 200, cutting height = 0.99, and deepSplit = F). The intramodular connectivity (Kwithin) was then used to characterize the hub genes in each module, which represents the strong gene connection to other genes in the module. Each node (gene) usually connected to many other nodes (genes) as the connection edges with different weight values. Moreover, the cold responsive modules (CRMs) were identified based on the over-representation of tissue-specific DEGs using a hypergeometric test (*p* < 0.05). Furthermore, GO terms were enriched for genes in each module as the functional annotation by EnrichPipeline [30]. Cytoscape was used to visualize the coexpression network, which filtered the edges and focused on the nodes (genes) with the strongest edges [31].

## 3. Results and Discussion

### 3.1. Histological Observation of P. olivaceus Tissues after Cold Shock Challenge

**Figure 1:**
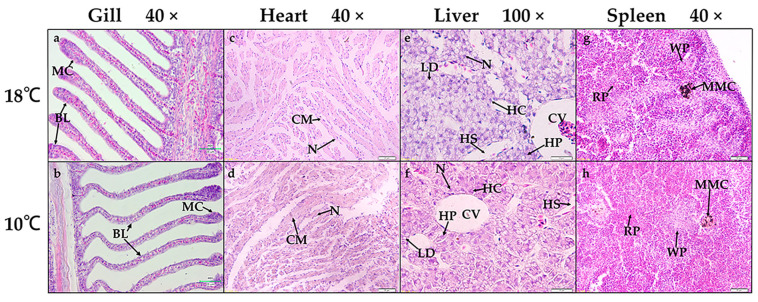
Effects of cold shock stress on *P. olivaceus* gill, heart, liver, and spleen tissues with H&E staining. (**a**,**b**) Gills, (**c**,**d**) hearts, (**e**,**f**) livers, and (**g**,**h**) spleens from control and cold shock groups. MC: mucous cell, BL: branchial lamellae, CM: cardiac muscle cell, N: nucleus, HS: hepatic sinusoid, HP: hepatic plate, HC: hepatocyte, LD: lipid drop, CV: central vein, MMC: melanin macrophage center, RP: red pulp, WP: white pulp.

After cold shock, there was a slight swelling of the lamellae and epithelial cells in *P. olivaceus* gills compared with those of the control group, and the supporting structure of the gill filament shrunk and the branchial lamellae were curlier (Figure 1a,b). This result was similar to that of four-finger threadfin (*Eleutheronema tetradactylum*) gills under cold shock, in which the branchial lamellae of *E. tetradactylum* shrunk, swelled, and curved [32]. There was no significant difference in the *P. olivaceus* hearts between the cold shock group and the control group (Figure 1c,d), which has also rarely been described in other fish species. In the livers, the hepatic sinusoids contracted and the gap between hepatocytes expanded under cold shock, which was possibly caused by the contraction of the hepatocytes (Figure 1e,f). There was also obvious shrinkage of and reduction in the lipid drops in the hepatic stellate cells, which suggested the consumption of fat under cold shock stress (Figure 1e,f). Under cold stress, morphological changes such as cavitation of the hepatocytes, contraction of the liver and hepatic blood sinuses, deepening of hepatocyte staining, severe damage and deformation of hepatocytes, as well as atrophy or disappearance of some hepatocyte nuclei, were also reported in the liver of *E. tetradactylum* [32]. Moreover, there was no significant difference in *P. olivaceus* spleens between the cold shock group and the control group (Figure 1g,h). Therefore, compared with at a medium temperature (18 °C), acute cold shock (10 °C) induced varying degrees of *P. olivaceus* tissue structure changes, especially in the gills and livers.

### 3.2. Tissue-Specific Transcriptomic Response of P. olivaceus under Cold Shock Stress

**Figure 2:**
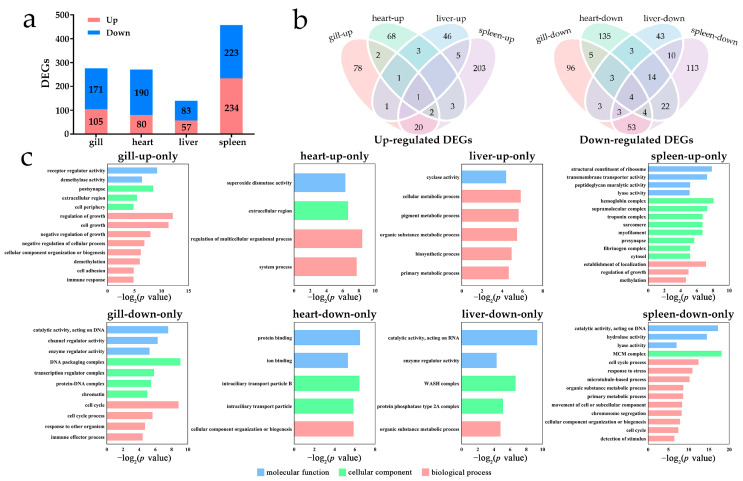
Differential gene expression in *P. olivaceus* gill, heart, liver, and spleen tissues under acute cold shock challenge. (**a**) Differentially expressed genes in the four tissues as |log_2_FC| > 1.5 and *q*-value < 0.05; (**b**) DEGs shown as the Venn diagrams among the four tissues; (**c**) Functional GO enrichment of the tissue-specific DEGs at level 3.

To characterize the transcriptomic response of *P*. *olivaceus* tissues under acute cold shock stress, 24 RNA-seq libraries from the gill, heart, liver, and spleen tissues were analyzed. Firstly, compared with the control group, a total of 276 (105 up- and 171 downregulated), 270 (80 up- and 190 downregulated), 140 (57 up- and 83 downregulated), and 457 (234 up- and 223 downregulated) differentially expressed genes (DEGs, |log_2_FC| ≥ 1.5 and *q* < 0.05) were identified in the gill, heart, liver, and spleen tissues, respectively (Figure 2a and Figure S1). There were more tissue-specific DEGs than the few overlapped DEGs among the challenged tissues (Figure 2b), with the gills and spleens sharing 20 upregulated and 53 downregulated DEGs (Figure 2b). For the overlapped DEGs, the single upregulated DEG for all the four tissues was *period circadian protein homolog 2* (PER2) and the four downregulated DEGs shared by the four tissues were *interleukin-10 receptor subunit beta* (Il10rb), *C-X-C motif chemokine 10* (CXCL10), *putative nuclease HARBI1* (HARBI1), and *serine/threonine-protein phosphatase* (PPP3CA). Moreover, the abundant tissue-specific DEGs indicated the particular gene sets participating in the diverse cellular processes in *P. olivaceus* tissues. Specifically, the tissue-specific upregulated DEGs were enriched in GO terms including “cell growth”, “demethylase activity”, “extracellular matrix,” and “immune response” in the gills; “circulatory system”, “oxidation”, “extracellular region”, and “cytoskeleton” in the hearts; “heme oxidation”, “lipase inhibitor activity”, and “cellular metabolic process” in the livers; and “transmembrane transport”, “cell cycle”, and “cell growth” in the spleens (Figure 2c and Table S1). The tissue-specific downregulated DEGs were mainly enriched in GO terms such as “cell cycle”, “response to stimulus”, “cell division”, and “chromatin” in the gills; “transport”, “cytoskeleton”, and “oxidation” in the hearts; “oxidation” and “lipid metabolism” in the livers; “cell cycle”, “response to stress”, “chromosome segregation”, and “microtubule-based process” in the spleens (Figure 2c and Table S1). These regulated DEGs indicated that under the cold shock challenge, significant tissue-specific cellular process alterations and stress effects occur in *P*. *olivaceus*.

### 3.3. Tissue Specific Coexpression Network of P. olivaceus DEGs under Cold Shock Stress

**Figure 3:**
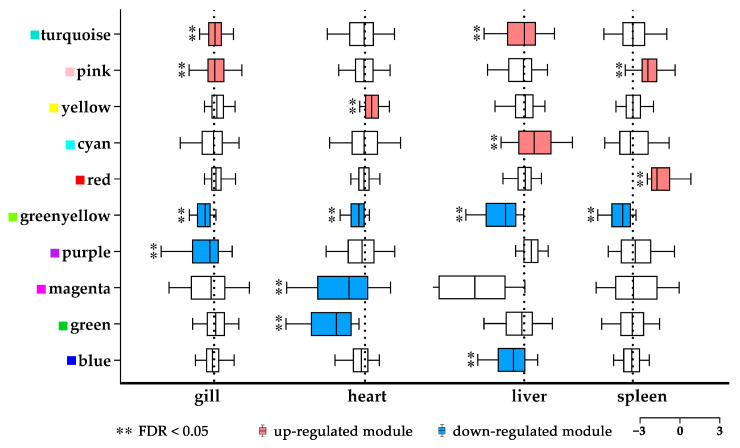
WGCNA in *P. olivaceus* tissues under acute cold shock stress. Cold responsive modules (CRMs) were identified through the enrichment analysis of DEGs with cold shock stress. Red and blue boxes indicate up- and downregulated modules, respectively.

To further understand the modulated gene regulation and interaction among tissues of *P. olivaceus* under cold shock stress, 20,877 expressed genes from the 24 tissue transcriptome samples were used to perform WGCNA, and they were assigned into 18 modules with a size ranging from 288 to 3613 genes (Figure 3 and Figure S2 and Table S2). The cold responsive modules (CRMs) were identified based on the over-representation of DEGs. As a result, 10 CRMs were identified (*FDR* < 0.05), including 5 modules enriching activated and inhibited DEGs each (Figure 3 and Table S3). Among the CRMs, two, one, two, and two modules showed enriched upregulated DEGs (*FDR* < 0.05) for gills (turquoise and pink), hearts (yellow), livers (turquoise and cyan), and spleens (pink and red), respectively; while two, three, two, and one modules were enriched in downregulated DEGs (*FDR* < 0.05) for gills (green-yellow and purple), hearts (green-yellow, magenta, and green), livers (green-yellow and blue), and spleens (green-yellow), respectively (Figure 3 and Table S3). Interestingly, among the 10 SRMs, 3 modules represented overlapped responsive pattern between the tissues, with the turquoise (gill and liver) and pink (gill and spleen) modules being upregulated, whereas the green-yellow module being downregulated for all four tissues (Figure 3). This could confirm the diversified regulatory patterns as revealed by the transcriptome analysis (Figure 2) among the *P. olivaceus* tissues under cold shock stress.

### 3.4. Coordinated Regulation of the Tissue-Specific P. olivaceus DEGs

**Figure 4:**
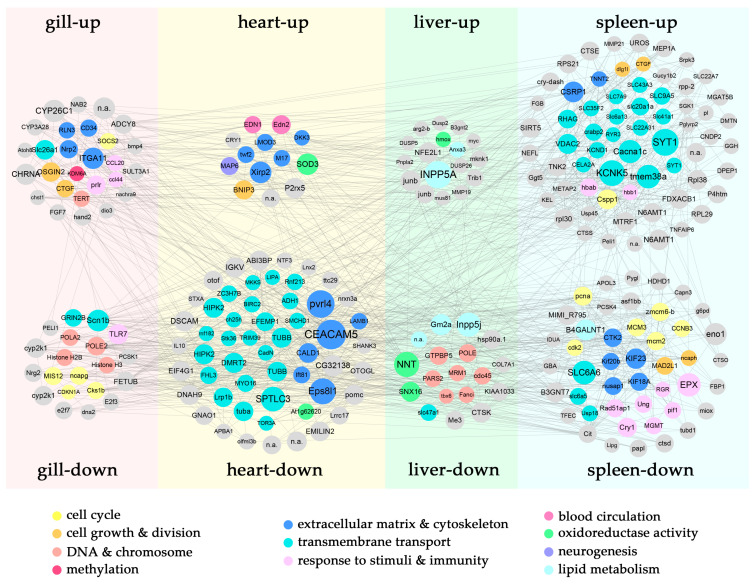
The tissue-specific gene networks of DEGs from the cold responsive modules in *P. olivaceus*. The genes were retrieved from the DEGs with enriched GO functions: the circle size represents the intramodular connectivity (Kwithin); the circle color represents the annotated GO functions.

**Figure 5:**
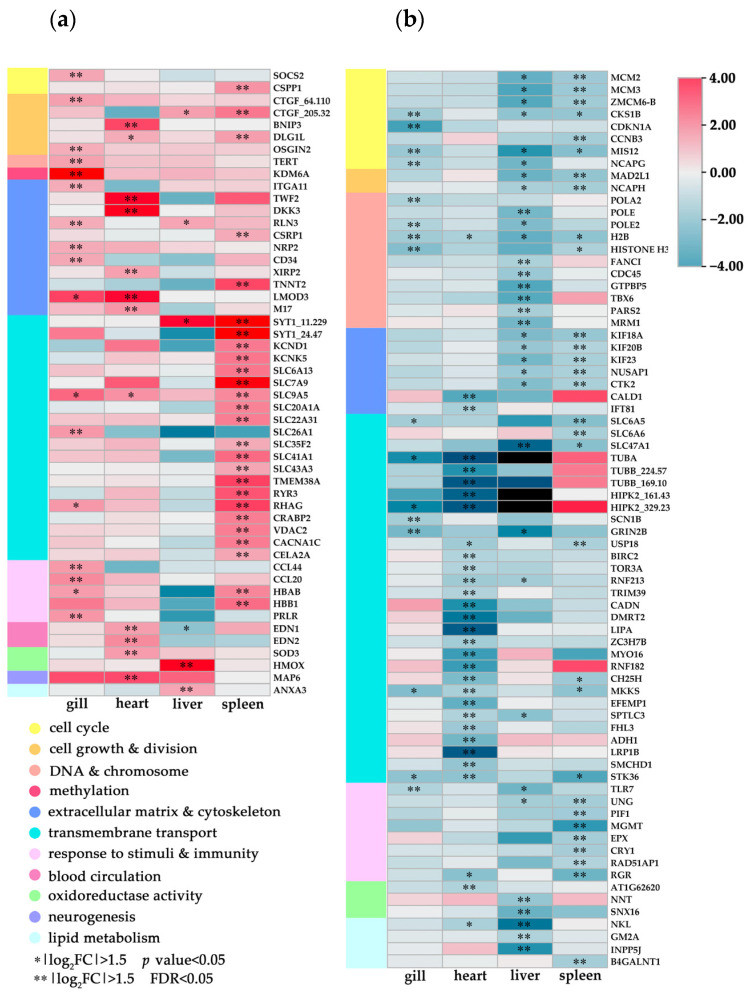
Differential expression of functional annotated genes in *P. olivaceus* tissues under acute cold stress. (**a**) Upregulated DEGs and (**b**) downregulated DEGs with their GO term clustering.

In the CRMs, many DEGs represented tissue-specific regulation under cold shock challenge (Figure 2b), which resulted in diverse cellular responses in *P. olivaceus* tissues. These DEGs from enriched GO functions (Table S4) were interlinked in the networks (Figure 4). Therefore, the CRMs and DEGs as key functional nodes were investigated and discussed to illustrate the core gene interactions in the tissue-specific responses of *P. olivaceus*. Firstly, several gill-specific DEGs were interlinked in the networks. For example, as an upregulated DEG, *neuropilin-2* (NRP2) could act as a receptor for entry into the epithelial and endothelial cells of the gills; *integrin alpha-11* (ITGA11) is the receptor for collagen; and *cell antigen CD34* is the adhesion molecule mediating the attachment of stem cells to the extracellular matrix (ECM) [33,34,35]. All these DEGs are from the “extracellular region” and “cell adhesion” functions (Figure 4 and Figure 5), as key mediators of cell–matrix and cell–cell adhesion, suggesting their essential roles in the ECM structure maintenance and cell matrix adhesion in the cellular homeostasis of *P. olivaceus* gill filaments and lamella under the cold shock stress. Moreover, the downregulated DEGs, mainly from the “cell cycle” and “cell division” functions, were interlinked in the gill-specific networks (Figure 4). For instance, *DNA polymerase* (POLA2 and POLE2) and *histone* (H2B and H3) play essential roles in transcription regulation, DNA repair, DNA replication, and chromosomal stability; *cyclin-dependent kinase inhibitor 1* (CDKN1A) may be involved in the p53/TP53-mediated inhibition of cellular proliferation in response to DNA damage; and protein *mis12* is required for normal chromosome alignment and segregation and for kinetochore formation during mitosis [36,37,38]. These interlinked gill-specific DEGs suggested cellular damage in the gill filaments and lamina under cold shock challenges, which could also be observed from the H&E staining (Figure 1a,b). Similar regulated functions were also identified in the gills of *P. olivaceus* under heat shock, in which the upregulated functions were mainly enriched in the cellular response to stimuli, protein refolding, and regulation of apoptotic process, while the downregulated functions were mostly enriched in DNA repair and replication, as well as the cell cycle [14]. Interestingly, cyclin-dependent protein kinase activity was identified in *P. olivaceus* gills under both heat [14] and cold stress, which may suggest that the downregulation of the cell-cycle function may be a common response in *P. olivaceus* gills under both heat and cold shock stresses.

In the heart-specific upregulated DEGs, cellular-structure-related functions such as “actin filament” and “cytoskeleton” were enriched. For example, *xin actin-binding repeat-containing protein 2* (XIRP2) can protect actin filaments from depolymerization; *leiomodin-3* (LMOD3) increases the rate of actin polymerization; and *microtubule-associated protein 6* (MAP6) specifically shows microtubule cold-stabilizing activity [39,40,41,42] (Figure 4 and Figure 5). All these DEGs indicated the activation of intracellular movements and membrane trafficking in the *P. olivaceus* heart, which could interact with the circulatory functional gene *endothelin* (EDN1, EDN2) to cope with cold stress (Figure 4). Moreover, *superoxide dismutase* (SOD3), which can protect the extracellular space from the toxic effects of reactive oxygen intermediates [43], was also interlinked in the network, suggesting antioxidative protection in the *P. olivaceus* cardiovascular system (Figure 4 and Figure 5). In addition, many transport and cytoskeleton-related downregulated DEGs were interlinked in the networks. For example, *tubulins* (TUBA and TUBB) are the major constituents of microtubules; *myosin* (Myo16) is an actin-based motor molecules with ATPase activity, which can serve in intracellular movements; *homeodomain-interacting protein kinase 2* (HIPK2) is involved in transcription regulation, p53/TP53-mediated cellular apoptosis, and regulation of the cell cycle [37,44,45,46] (Figure 4 and Figure 5). These heart-specific DEGs indicated that even with fine protection, cellular injury could still be found in *P. olivaceus* heart under cold stress. A sharp temperature decline can lead to impaired cardiac contractile function in fish, as the thin filaments in the cardiac muscle are less sensitive to Ca^2+^ at lower temperatures [47]. For example, when compared at the respective physiological temperature and pH, rainbow trout (*Oncorhynchus mykiss*) cardiac actin-myosin ATPase activity showed more Ca^2+^ sensitivity than that of rats, which may help *O. mykiss* to offset the cardioplegic effects of cold [47]. Moreover, after cold acclimation, myocardial fibrillary collagen content and/or connective tissue content increases, which may protect the myocardium from the increased hemodynamic stress due to pumping cold viscous blood [47].

There were fewer liver-specific DEGs in *P. olivaceus* livers than in other tissues from the network (Figure 2b and 4). For example, of the liver-specific upregulated DEGs, *heme oxygenase* (HMOX) catalyzes the oxidative cleavage of heme, which can protect against cold-injury-induced cell loss and damage [48], while in the downregulated DEGs, *DNA polymerase* (POLE) and *cell division control protein* (CDC45) are both required for the initiation of chromosomal DNA replication. *Mitochondrial ribosome-associated GTPase 2* (GTPBP5) plays a role in the regulation of the mitochondrial ribosome assembly and of translational activity, all suggesting the inhibited function of the “cell cycle” and “cell division” [49,50,51,52]. In the livers of fish, high-temperature stress may affect apoptosis, DNA replication, protein metabolism, energy metabolism, lipid metabolism, immune, cell cycle, protein processing, and transport, as well as antioxidative responses [53,54,55], while low-temperature stress may affect signaling transduction, membrane fluidity, lipid metabolism, antioxidative responses, ion binding, macromolecule catabolism, mitochondrial enzymes related to transport, carbohydrate metabolism, and cell cycle endocrine system [56,57]. Interestingly, *inositol polyphosphate-5-phosphatase A* (INPP5A) and *phosphatidylinositol 4,5-bisphosphate 5-phosphatase A* (INPP5J) were interlinked as the up- and downregulated DEGs in the network, respectively (Figure 4 and Figure 5), which may suggest the effects of membrane fluidity and lipid metabolism involved in the modulation of inositol function and the influence of cell migration, adhesion, and polarity in *P. olivaceus* livers under cold shock [58].

Moreover, several spleen-specific upregulated DEGs interlinked in the networks were membrane- and (ion)-transporter activity-related genes (Figure 4), such as *solute carrier transporters* (SLC6a13, SLC7A9, SLC9A5, SLC20a1a, SLC22A31, SLC35F2, SLC41a1, and SLC43A3) (Figure 4 and Figure 5), which indicated the diverse functions of *SLCs* in transmembrane transport [59]. In addition, cell-cycle- and DNA-replication-related functions were inhibited in the downregulated spleen-specific DEGs. For example, *DNA replication licensing factors* (MCM2 and MCM3), *G2/mitotic-specific cyclin-B* (CCNB3), *kinesin-like proteins* (KIF18A, KIF20B, and KIF23), and *mitotic spindle assembly checkpoint protein* (MAD2A) can prevent the onset of anaphase until all chromosomes are properly aligned at the metaphase plate [60,61,62]. *Proliferating cell nuclear antigen* (PCNA) is also involved in the control of eukaryotic DNA replication by increasing the polymerase’s processability during elongation of the leading strand [50,63] (Figure 4 and Figure 5). There are only limited studies focused on fish spleen tissues under temperature challenges. For example, in the spleen of Nile tilapia (*Oreochromis niloticus*), immune and antioxidative process were affected, and xanthine oxidase, peroxidase, catalase, and superoxide dismutase played an important role [64]. Therefore, the strongly activated transport and inhibited cell cycle functions in the spleens revealed the severe effects of cold shock on *P. olivaceus* spleens, which may warrant further investigation of their specific functions.

### 3.5. Modulation of P. olivaceus CRMs with the Cold Shock Response

**Figure 6:**
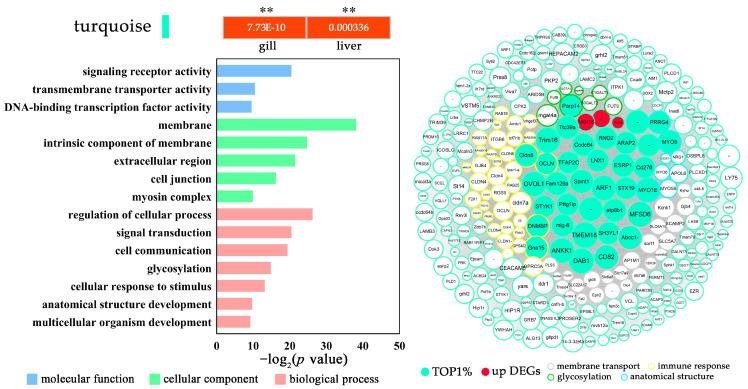
The GO enrichment (level 3) and gene network of the gill and liver upregulated turquoise module in *P. olivaceus* under acute cold shock stress. The network represents the top 300 genes with high connectivity. The circle size indicates the intramodular connectivity (Kwithin), and the color of the circle frame indicates the annotated GO terms. ** *p* < 0.01.

In addition to the DEGs functioning in coordination in the networks, many more non-DEGs, with known and novel functions, were also clustered in the CRMs (Table S4). To further investigate the tissue-specific modulated gene interaction in the given modules, the genes in each CRMs were annotated with GO (Table S4), and the functions of CRMs from overlapped tissues were characterized. For example, the turquoise module was mainly represented with gill- and liver upregulated genes and enriched functions including “membrane”, “extracellular matrix”, “cell junction”, “response to stimulus”, and “glycosylation” (Figure 6 and Table S4), indicating the activated ECM and stress gene regulation in *P. olivaceus* gills and livers under cold shock stress. More specifically, several *claudins* (CLDN1, CLDN4, CLDN6, CLDN7a, and CLDN8) and *occludin* (OCLN) genes were clustered as hub genes in the network (Figure 6)**,** which may play an essential role in the formation and regulation of the tight junction through calcium-independent cell-adhesion activity [65]. It was reported that cold exposure increases intestinal paracellular permeability to nutrients in mice, which was associated with a transient increase in the expression of CLDN2 [66]. In addition, *myosins* (MYO1E, MYO5B, MYO6) are actin-based motor molecules serve in intracellular movements; *epithelial splicing regulatory protein 1* (ESPR1) regulates the formation of epithelial-cell-specific isoforms; and *syntaxin-19* (STX19) plays a role in endosomal trafficking of the epidermal growth factor receptor [67,68,69]. These hub genes were also correlated in the network from the cytoskeleton and anatomical structure functions (Figure 6), which, together with CLDNs and OCLNs, could contribute to the maintenance of the cellular structure of *P. olivaceus* gills and livers under cold stress (Figure 1).

**Figure 7:**
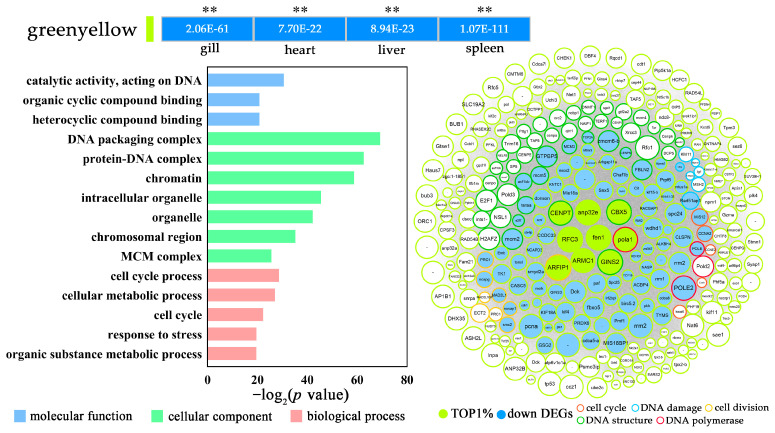
The GO enrichment (level 3) and gene network of the downregulated green-yellow module in *P. olivaceus* under acute cold shock stress. The network represents the top 300 genes with their high connectivity. The circle size indicates the intramodular connectivity (Kwithin), and the color of the circle frame indicates the annotated GO terms. ** *p* < 0.01.

More interestingly, the green-yellow module, representing the downregulated DEGs from all four tissues, was enriched in functions mainly including “cell cycle”, “cell division”, “DNA package complex”, “chromosomal region”, and “response to stress” (Figure 7 and Table S4), indicating the severe cellular function inhibition in *P. olivaceus* tissues under cold shock stress. For example, several *DNA polymerases* (POLA1, POLD2/3, POLE/E2, and POLE2) and *DNA replication complex GINS proteins* (GINS2, GINS3), both playing essential roles in the initiation of DNA synthesis [36,50], were the top hub genes from the “cell cycle” and “DNA complex” functions (Figure 7 and Figure S3). In addition, *G2/mitotic-specific cyclins* (CCNA2 and CCNE2)**,** interacting with *cyclin-dependent kinase* (CDK), are important for the control of the cell cycle at the G2/M (mitosis) transition [60]. Moreover, *cell division cycle associated family proteins* (CDCA5 and CDCA8) play a vital role in efficient DNA double-stranded break repair; *DNA replication licensing factors* (MCM2, MCM3, MCM4, and MCM5) are the replicative helicases essential for DNA replication initiation and elongation in eukaryotic cells; *kinesin-like proteins* (KIF4, KIF15, and KIF18) play a role in chromosome segregation during mitosis; and protein *mises* (MIS12, MIS18a, and MIS18BP) are required for normal chromosome alignment and segregation [38,61,70,71,72]. All these genes were the key hub genes mainly from the “cell cycle process” and “DNA structure/polymerase” functions (Figure 7 and Figure S3), suggesting severe cellular function inhibition among *P. olivaceus* tissues under cold shock. It was reported that many types of mammalian cells failed to undergo the G(2)/M transition after cooling from 37 °C to 16–20 °C, while the progress at G(1)/S was not affected by the same temperatures [73]. Moreover, in some mammalian cultures, the exposure to 4–10 °C can cause a high abundance of mitotic synchrony (up to 80%), which may involve the cell cycle checkpoint in response to cold shock stress, inhibiting the G(1)/S transition [73]. In teleost fish under temperature changes, cell cycle progress is also regulated in *P. olivaceus* gills and livers under heat shock [14,53], and cold-responsive genes in zebrafish (*Danio rerio*) and common carp (*Cyprinus carpio*) under cold stress are mostly involved in oxidative phosphorylation, protein folding and degradation, RNA processing, and translation, which comprise the set of evolutionarily conserved cold-responsive mechanisms in teleosts [10]. Therefore, in this study, the CDC45-MCM-GINS (CMG) helicase, the molecular machine that unwinds template DNA during replication, could be strongly inhibited among *P. olivaceus* gill, heart, liver and spleen tissues during the cold shock, which warrants comprehensive investigation of their functions in further studies.

## 4. Conclusions

The poikilothermal teleost species present diverse temperature responses reflected in the tissue-specific cellular, antioxidative and immune statuses. This study performed a systematic transcriptomic and gene coexpression network survey in Japanese flounder (*P. olivaceus*) tissues under cold shock stress. The tissue-specific modulated expression regulation of genes was observed in response to acute cold shock, which was mainly found in the activated “extracellular matrix” and “cytoskeleton” functions, as well as in the inhibited “cell cycle” and “DNA complex” functions in all tested tissues. These findings indicated that even with different levels of tissue-specific responses, cold shock may induce severely disrupted cellular functions in many *P. olivaceus* tissues, which reduce aquaculture productivity. Therefore, further investigation into the functions of specific CRM networks will provide more comprehensive insights into the cold adaptation mechanisms and will enable better conservation and cultivation of *P. olivaceus* in cold water.

## Data Availability

The *P. olivaceus* transcriptome datasets used in this study can be found in the NCBI Sequence Read Archive (SRA) BioProject PRJNA717098, PRJNA717103, PRJNA717106, and PRJNA717107.

## Supplementary Materials

The following supporting information can be downloaded at: https://www.mdpi.com/article/10.3390/biology12060784/s1. Figure S1: Volcano plots for the DEGs of the four *P. olivaceus* tissues under cold shock; Figure S2: Modulation from the WGCNA; Figure S3: Expression profiles of the genes enriched in the cell-cycle-related functions in the four *P. olivaceus* tissues under cold shock; Table S1: GO enrichment of the tissue-specific DEGs from the four *P. olivaceus* tissues under cold shock; Table S2: General information for the modules of WGCNA; Table S3: Identification of CRMs; Table S4: GO enrichment of each CRM.

## References

[1] Alfonso S., Gesto M., Sadoul B. (2021). Temperature increase and its effects on fish stress physiology in the context of global warming. J. Fish. Biol..

[2] Reid C.H., Patrick P.H., Rytwinski T., Taylor J.J., Willmore W.G., Reesor B., Cooke S.J. (2022). An updated review of cold shock and cold stress in fish. J. Fish. Biol..

[3] Donaldson M.R., Cooke S.J., Patterson D.A., Macdonald J.S. (2010). Cold shock and fish. J. Fish. Biol..

[4] Cheng C.H., Liang H.Y., Luo S.W., Wang A.L., Ye C.X. (2017). The protective effects of vitamin C on apoptosis, DNA damage and proteome of pufferfish (*Takifugu obscurus*) under low temperature stress. J. Therm. Biol..

[5] Chandra J., Samali A., Orrenius S. (2000). Triggering and modulation of apoptosis by oxidative stress. Free. Radic. Biol. Med..

[6] Ren X., Yu X., Gao B., Liu P., Li J. (2017). Characterization of three caspases and their pathogen-induced expression pattern in *Portunus trituberculatus*. Fish. Shellfish. Immun..

[7] Zhi B., Wang L., Wang G., Zhang X. (2011). Contribution of the caspase gene sequence diversification to the specifically antiviral defense in invertebrate. PLoS ONE.

[8] Nie M., Hu J., Lu Y., Wu Z., Wang L., Xu D., Zhang P., You F. (2019). Cold effect analysis and screening of SNPs associated with cold-tolerance in the olive flounder *Paralichthys olivaceus*. J. Appl. Ichthyol..

[9] Nie M., Lu Y., Zou C., Wang L., Zhang P., You F. (2020). Insight into AMPK regulation mechanism *in vivo* and *in vitro*: Responses to low temperatures in the olive flounder *Paralichthys olivaceus*. J. Thermal Biol..

[10] Hu P., Liu M., Zhang D., Wang J., Niu H., Liu Y., Wu Z., Han B., Zhai W., Shen Y. (2015). Global identification of the genetic networks and cis-regulatory elements of the cold response in zebrafish. Nucleic Acids Res..

[11] Gao S., Chang Y., Zhao X., Sun B., Zhang L., Liang L., Dong Z. (2020). The effect of different bicarbonate alkalinity on the gill structure of amur ide (*Leuciscus waleckii*). Acta Hydrobiol. Sinica..

[12] Vornanen M., Hassinen M., Koskinen H., Krasnov A. (2005). Steady-state effects of temperature acclimation on the transcriptome of the rainbow trout heart. Am. J. Physiol. Regul. Integr. Comp. Physiol..

[13] Liu Q., Wen J., Ou Y., Li J., Zhou H. (2017). Effects of transport stress on liver, gill and spleen tissue structure of juvenile *Eleutheronema tetradactylum*. J. South. Agric..

[14] Yan W., Qiao Y., He J., Qu J., Liu Y., Zhang Q., Wang X. (2022). Molecular mechanism based on histopathology, antioxidant system and transcriptomic profiles in heat stress response in the gills of Japanese flounder. Int. J. Mol. Sci..

[15] Quiring L., Walter B., Lohaus N., Schwan D., Rech A., Dlugos A., Rauen U. (2022). Characterisation of cold-induced mitochondrial fission in porcine aortic endothelial cells. Mol. Med..

[16] Pucci F., Rooman M. (2017). Physical and molecular bases of protein thermal stability and cold adaptation. Curr. Opin. Struct. Biol..

[17] Schleger I.C., Pereira D.M.C., Resende A.C., Romão S., Herrerias T., Neundorf A.K.A., Sloty A.M., Guimarães I.M., de Souza M.R.D.P., Carster G.P. (2022). Cold and warm waters: Energy metabolism and antioxidant defenses of the freshwater fish *Astyanax lacustris* (*Characiformes:Characidae*) under thermal stress. J. Comp. Physiol. B.

[18] Langfelder P., Horvath S. (2008). WGCNA: An R package for weighted correlation network analysis. BMC Bioinform..

[19] Zhu Q., Li M., Lu W., Wang Y., Li X., Cheng J. (2023). Transcriptomic modulation reveals the specific cellular response in Chinese sea bass (*Lateolabrax maculatus*) gills under salinity change and alkalinity stress. Int. J. Mol. Sci..

[20] Yang Y., Liu Q., Xiao Y., Xu S., Wang X., Yang J., Song Z., You F., Li J. (2019). High temperature increases the *gsdf* expression in masculinization of genetically female Japanese flounder (*Paralichthys olivaceus*). Gen. Comp. Endocrinol..

[21] Yamaguchi T., Kitano T. (2012). High temperature induces *cyp26b1* mRNA expression and delays meiotic initiation of germ cells by increasing cortisol levels during gonadal sex differentiation in Japanese flounder. Biochem. Biophys. Res. Commun..

[22] Lu Y., Wu Z., Song Z., Xiao P., Liu Y., Zhang P., You F. (2016). Insight into the heat resistance of fish via blood: Effects of heat stress on metabolism, oxidative stress and antioxidant response of olive flounder *Paralichthys olivaceus* and turbot *Scophthalmus maximus*. Fish. Shellfish. Immunol..

[23] Xiang Y., Zhang J., Shi Z. (2018). Effects of high temperature and sex hormone in expression of *kiss2* and *gpr54-2* genes in olive flounder *Paralichthys olivaceus*. Fish. Sci..

[24] Hu J., You F., Wang Q., Weng S., Liu H., Wang L., Zhang P.J., Tan X. (2014). Transcriptional responses of olive flounder (*Paralichthys olivaceus*) to low temperature. PLoS ONE.

[25] Kurlta Y., Sakuma T., Kakehi S., Shimamura S., Sanematsu A., Kitagawa H., Ito S.I., Kawabe R., Shibata Y., Tomiyama T. (2021). Seasonal changes in depth and temperature of habitat for Japanese flounder *Paralichthys olivaceus* on the Pacific coast of northeastern Japan. Fish. Sci..

[26] Xin M., Ma D., Wang B. (2015). Chemicohydrographic characteristics of the Yellow Sea Cold Water Mass. Acta Oceanol. Sin..

[27] Lu Y., Nie M., Wang L., Xiong Y., Wang F., Wang L., Xiao P., Wu Z., Liu Y., You F. (2018). Energy response and modulation of AMPK pathway of the olive flounder *Paralichthys olivaceus* in low-temperature challenged. Aquaculture.

[28] Pertea M., Kim D., Pertea G.M., Leek J.T., Salzberg S.L. (2016). Transcript-level expression analysis of RNA-seq experiments with HISAT, StringTie and Ballgown. Nat. Protoc..

[29] Chen C., Chen H., Zhang Y., Thomas H.R., Frank M.H., He Y., Xia R. (2020). TBtools: An integrative toolkit developed for interactive analyses of big biological data. Mol. Plant..

[30] Chen S., Yang P., Jiang F., Wei Y., Ma Z., Kang L. (2010). De novo analysis of transcriptome dynamics in the migratory locust during the development of phase traits. PLoS ONE.

[31] Shannon P., Markiel A., Ozier O., Baliga N.S., Wang J.T., Ramage D., Amin N., Schwikowski B., Ideker T. (2003). Cytoscape: A software environment for integrated models of biomolecular interaction networks. Genome Res..

[32] Liu Q. (2017). The Effects of Operation and Low Temperature Stress on Organization Structure, Antioxidant System of Juvenile *Eleutheronema tetradactylum*. Master’s Thesis.

[33] Gerna G., Kabanova A., Lilleri D. (2019). Human cytomegalovirus cell tropism and host cell receptors. Vaccines.

[34] Koivunen J., Tu H., Kemppainen A., Anbazhagan P., Finnilä M.A., Saarakkala S., Käpylä J., Lu N., Heikkinen A., Juffer A.H. (2021). Integrin α11β1 is a receptor for collagen XIII. Cell Tissue Res..

[35] Zhang S., Ma X., Guo J., Yao K., Wang C., Dong Z., Zhu H., Fan F., Huang Z., Yang X. (2017). Bone marrow CD34+ cell subset under induction of moderate stiffness of extracellular matrix after myocardial infarction facilitated endothelial lineage commitment *in vitro*. Stem Cell Res. Ther..

[36] Wang C., Huang J., Li Y., Zhang J., He C., Li T., Jiang D., Dong A., Ma H., Copenhaver G.P. (2022). DNA polymerase epsilon binds histone H3.1-H4 and recruits MORC1 to mediate meiotic heterochromatin condensation. Proc. Natl. Acad. Sci. USA.

[37] Suzuki N., Johmura Y., Wang T.W., Migita T., Wu W., Noguchi R., Yamaguchi K., Furukawa Y., Nakamura S., Miyoshi I. (2021). TP53/p53-FBXO22-TFEB controls basal autophagy to govern hormesis. Autophagy.

[38] Ladurner R., Straight A.F. (2016). MIS12/MIND control at the kinetochore. Cell.

[39] Nilsson M.I., Nissar A.A., Al-Sajee D., Tarnopolsky M.A., Parise G., Lach B., Fürst D.O., van der Ven P.F.M., Kley R.A., Hawke T.J. (2013). Xin is a marker of skeletal muscle damage severity in myopathies. Am. J. Pathol..

[40] Lin F.H., Wang A., Dai W., Chen S., Ding Y., Sun L.V. (2020). Lmod3 promotes myoblast differentiation and proliferation via the AKT and ERK pathways. Exp. Cell Res..

[41] Flores-Martin J.B., Bonnet L.V., Palandri A., Zamanillo-Hermida S., Hallak M.E., Galiano M.R. (2022). The 19S proteasome subunit Rpt5 reversibly associates with cold-stable microtubules in glial cells at low temperatures. FEBS Lett..

[42] Delphin C., Bouvier D., Seggio M., Couriol E., Saoudi Y., Denarier E., Bosc C., Valiron O., Bisbal M., Arnal I. (2012). MAP6-F is a temperature sensor that directly binds to and protects microtubules from cold-induced depolymerization. J. Biol. Chem..

[43] Zelko I.N., Mariani T.J., Folz R.J. (2002). Superoxide dismutase multigene family: A comparison of the CuZn-SOD (SOD1), Mn-SOD (SOD2), and EC-SOD (SOD3) gene structures, evolution, and expression. Free. Radic. Biol. Med..

[44] Roll-Mecak A. (2020). The tubulin code in microtubule dynamics and information encoding. Dev. Cell.

[45] Bugyi B., Kengyel A. (2020). Myosin XVI. Adv. Exp. Med. Biol..

[46] Wook Choi D., Yong Choi C. (2014). HIPK2 modification code for cell death and survival. Mol. Cell Oncol..

[47] Keen A.N., Klaiman J.M., Shiels H.A., Gillis T.E. (2017). Temperature-induced cardiac remodelling in fish. J. Exp. Biol..

[48] Shih R.H., Cheng S.E., Tung W.H., Yang C.M. (2010). Up-regulation of heme oxygenase-1 protects against cold injury-induced brain damage: A laboratory-based study. J. Neurotrauma..

[49] Dai Y., Fleischhacker A.S., Liu L., Fayad S., Gunawan A.L., Stuehr D.J., Ragsdale S.W. (2022). Heme delivery to heme oxygenase-2 involves glyceraldehyde-3-phosphate dehydrogenase. Biol. Chem..

[50] Labib K., Gambus A. (2007). A key role for the GINS complex at DNA replication forks. Trends Cell Biol..

[51] Zou L., Mitchell J., Stillman B. (1997). CDC45, a novel yeast gene that functions with the origin recognition complex and MCM proteins in initiation of DNA replication. Mol. Cell. Biol..

[52] Cipullo M., Pearce S.F., Lopez Sanchez I.G., Gopalakrishna S., Krüger A., Schober F., Busch J.D., Li X., Wredenberg A., Atanassov I. (2021). Human GTPBP5 is involved in the late stage of mitoribosome large subunit assembly. Nucleic Acids Res..

[53] Qiao Y. (2022). Molecular Mechanism of Liver Response to High Temperature stress in Japanese Flounder (*Paralichthys olivaceus*). Master Thesis.

[54] Li Y. (2018). Transcriptome Analysis of Liver and Head Kidney in Rainbow Trout (*Oncorhynchus mykiss*) Reveals the Response to Heat Stress by RNA-seq. Ph.D. Thesis.

[55] Wei L. (2020). Transcriptome analysis of brain and liver in Nile tilapia (*Oreochromis niloticus*) reveals the response to high temperature by RNA-seq. Master’s Thesis.

[56] Miao B.B., Niu S.F., Wu R.X., Liang Z.B., Tang B.G., Zhai Y., Xu X.Q. (2021). Gene expression profile and co-expression network of pearl gentian grouper under cold stress by integrating Illumina and PacBio sequences. Animals.

[57] Wen X., Chu P., Xu J., Wei X., Fu D., Wang T., Yin S. (2021). Combined effects of low temperature and salinity on the immune response, antioxidant capacity and lipid metabolism in the pufferfish (*Takifugu fasciatus*). Aquaculture.

[58] Ramos A.R., Elong Edimo W., Erneux C. (2018). Phosphoinositide 5-phosphatase activities control cell motility in glioblastoma: Two phosphoinositides PI(4,5)P2 and PI(3,4)P2 are involved. Adv. Biol. Regul..

[59] César-Razquin A., Snijder B., Frappier-Brinton T., Isserlin R., Gyimesi G., Bai X., Reithmeier R.A., Hepworth D., Hediger M.A., Edwards A.M. (2015). A call for systematic research on solute carriers. Cell.

[60] Otto T., Sicinski P. (2017). Cell cycle proteins as promising targets in cancer therapy. Nat. Rev. Cancer..

[61] Pasion S.G., Forsburg S.L. (2001). Deconstructing a conserved protein family: The role of MCM proteins in eukaryotic DNA replication. Genet. Eng..

[62] Kim J.H., Kim H.R., Patel R. (2023). Inactivation of Mad2B enhances apoptosis in human cervical cancer cell line upon cisplatin-induced DNA damage. Biomol. Ther..

[63] González-Magaña A., Blanco F.J. (2020). Human PCNA structure, function and interactions. Biomolecules.

[64] Peng T., Hu T., Lin Y., Tang Z., Zeng L. (2012). Effects of low temperature stress on indices of biochemistry, immunity and antioxidation in Nile Tilapia. Fish. Sci..

[65] Barmeyer C., Schulzke J.D., Fromm M. (2015). Claudin-related intestinal diseases. Semin. Cell Dev. Biol..

[66] Price E.R., Ruff L.J., Guerra A., Karasov W.H. (2013). Cold exposure increases intestinal paracellular permeability to nutrients in the mouse. J. Exp. Biol..

[67] Masters T.A., Kendrick-Jones J., Buss F. (2017). Myosins: Domain organisation, motor properties, physiological roles and cellular functions. Handb. Exp. Pharmacol..

[68] Lu X., Li R., Wang X., Guo Q., Wang L., Zhou X. (2022). Overexpression of epithelial splicing regulatory protein 1 in metastatic lesions of serous ovarian carcinoma correlates with poor patient prognosis. Cancer Biother. Radiopharm..

[69] Ampah K.K., Greaves J., Shun-Shion A.S., Asnawi A.W., Lidster J.A., Chamberlain L.H., Collins M.O., Peden A.A. (2018). S-acylation regulates the trafficking and stability of the unconventional Q-SNARE STX19. J. Cell Sci..

[70] Zhang W., Qiu X., Sun D., Zhang D., Qi Y., Li X., Liu B., Liu J., Lin B. (2020). Systematic analysis of the clinical relevance of cell division cycle associated family in endometrial carcinoma. J. Cancer..

[71] Heath C.M., Wignall S.M. (2019). Chromokinesin Kif4 promotes proper anaphase in mouse oocyte meiosis. Mol. Biol. Cell..

[72] Spiller F., Medina-Pritchard B., Abad M.A., Wear M.A., Molina O., Earnshaw W.C., Jeyaprakash A.A. (2017). Molecular basis for Cdk1-regulated timing of Mis18 complex assembly and CENP-A deposition. EMBO Rep..

[73] Rieder C.L., Cole R.W. (2002). Cold-shock and the mammalian cell cycle. Cell Cycle..

