# Supplementary figures and images for: Transcriptomic Networks Reveal the Tissue-Specific Cold Shock Responses in Japanese Flounder (Paralichthys olivaceus)
Source: Biology (Basel). 2023 May 28;12(6):784. doi: 10.3390/biology12060784 (PMC10295398; doi:10.3390/biology12060784)

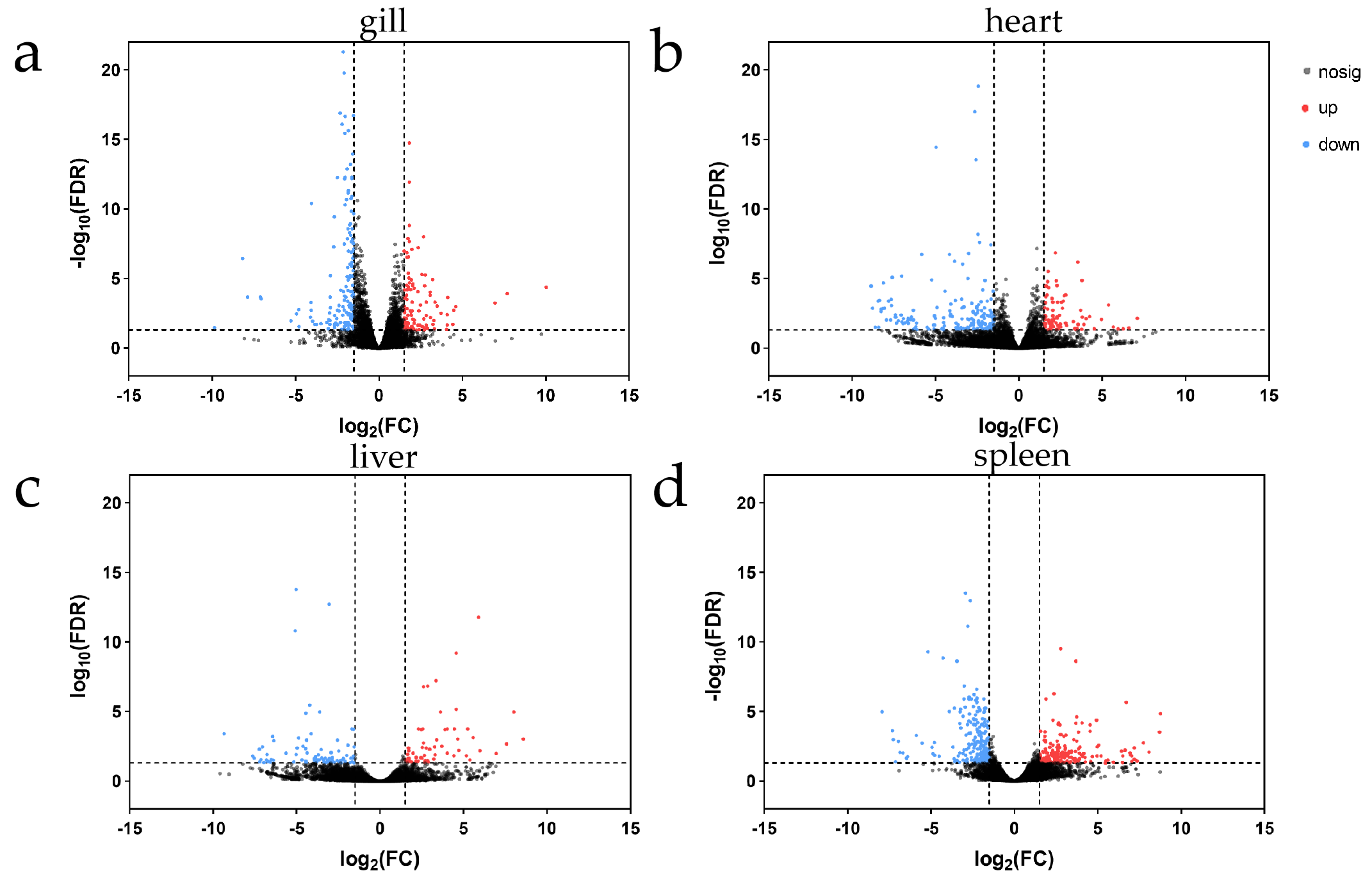

Supplement: Supplementary file 1 [file biology-12-00784-s001.zip › Fig.S1.Volcano.╞┤║╧(╨í).tif]

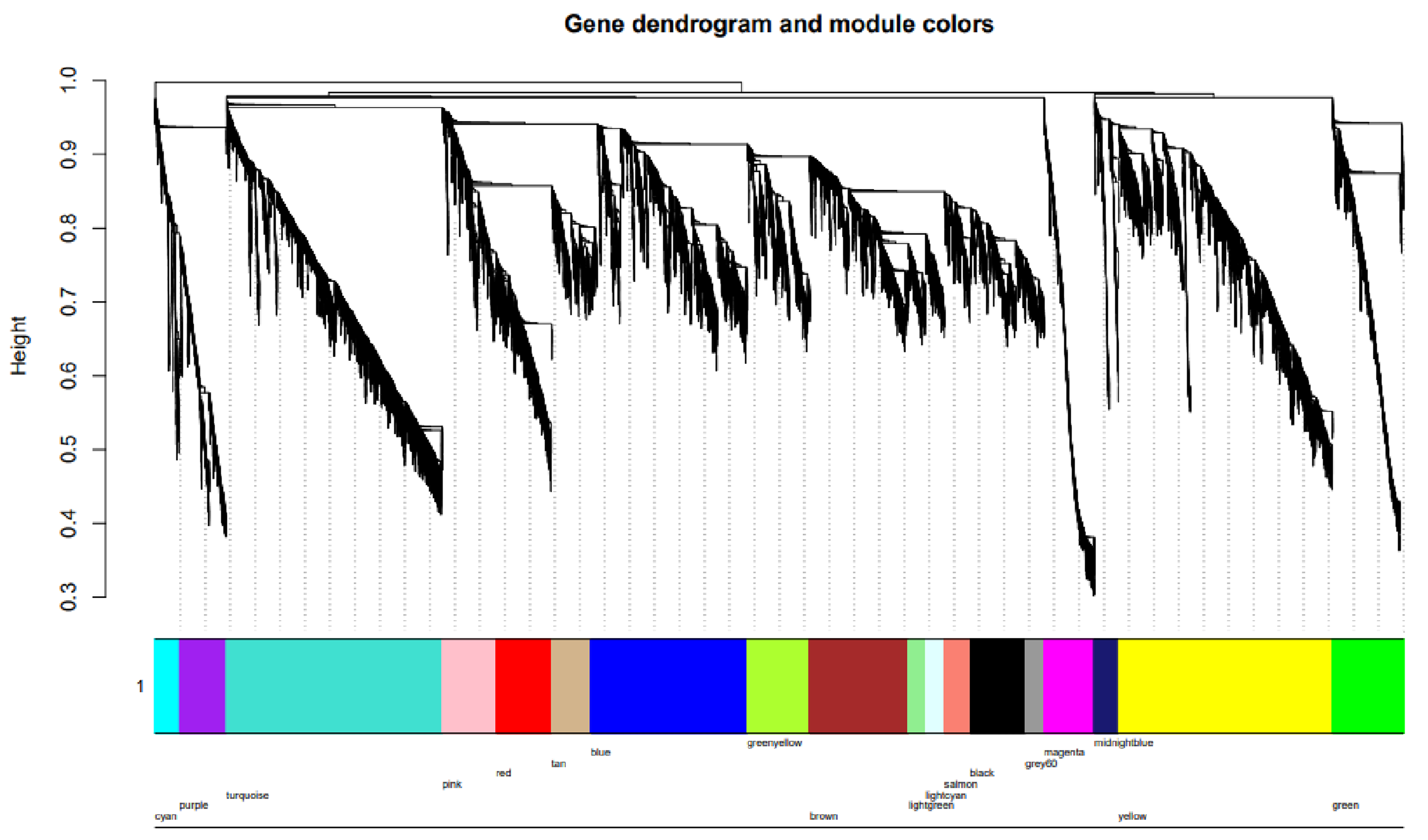

Supplement: Supplementary file 1 [file biology-12-00784-s001.zip › Fig.S2.WGCNA.tif]

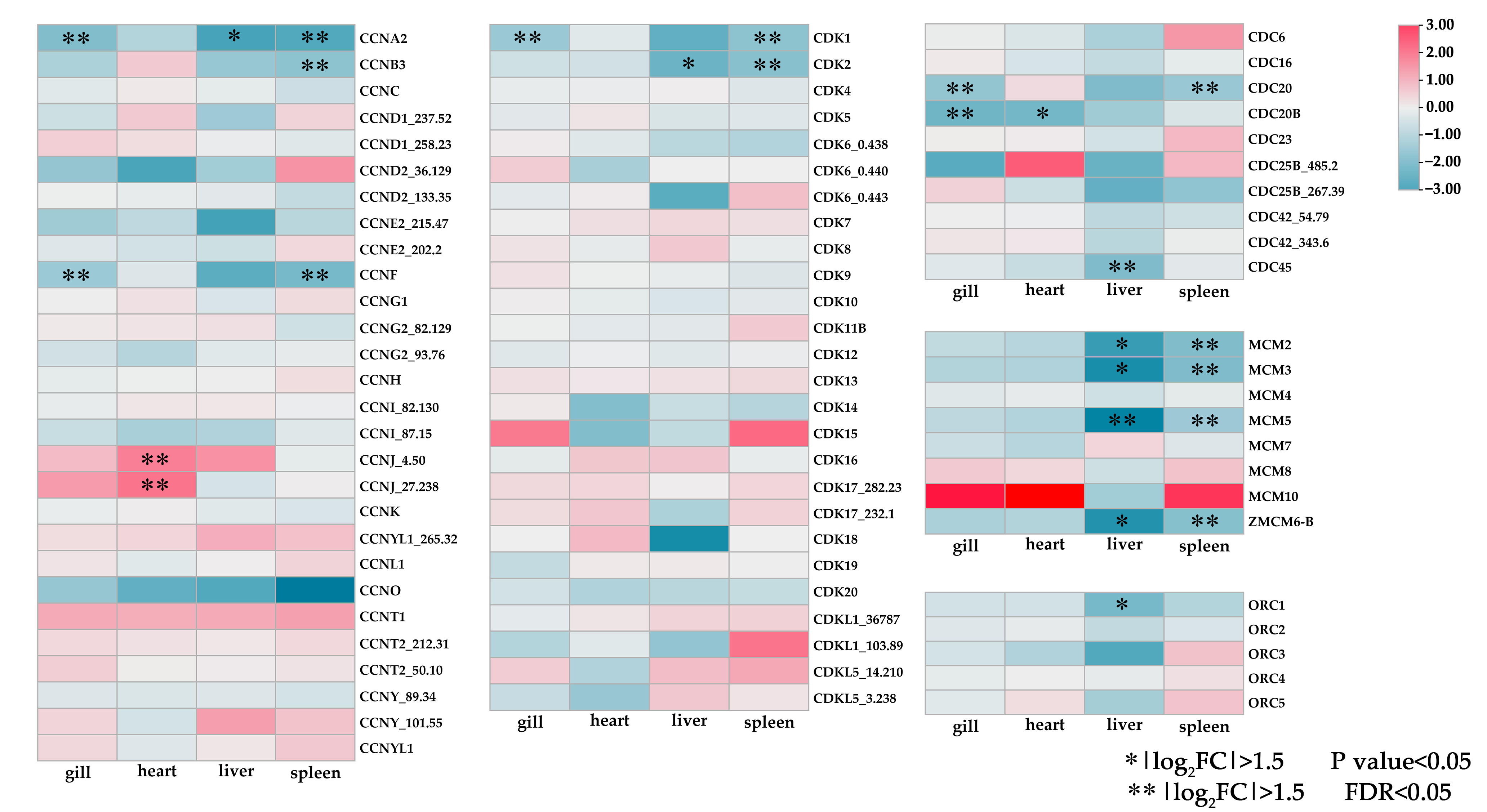

Supplement: Supplementary file 1 [file biology-12-00784-s001.zip › Fig.S3.CellCycle Heatmap+p-new.tif]
